# Surgical treatment of Epstein-Barr virus–associated lymphoepithelioma-like carcinoma occurring in both the posterior mediastinum and liver: Case report

**DOI:** 10.1097/MD.0000000000023610

**Published:** 2020-12-24

**Authors:** Xiao-Hui Qian, Dong-Kai Zhou, Wei-Lin Wang

**Affiliations:** a Department of Hepatobiliary and Pancreatic Surgery, The Second Affiliated Hospital, Zhejiang University School of Medicine; b Key Laboratory of Precision Diagnosis and Treatment for Hepatobiliary and Pancreatic Tumor of Zhejiang Province; c Research Center of Diagnosis and Treatment Technology for Hepatocellular Carcinoma of Zhejiang Province; d Clinical Medicine Innovation Center of Precision Diagnosis and Treatment for Hepatobiliary and Pancreatic Disease of Zhejiang University; e Clinical Research Center of Hepatobiliary and Pancreatic Diseases of Zhejiang Province, Hangzhou, China.

**Keywords:** case report, lymphoepithelioma-like carcinoma, positron emission tomography/computed tomography, posterior mediastinum, surgery

## Abstract

**Rationale:**

Lymphoepithelioma-like carcinoma (LELC) is a rare malignant tumor that can occur in many areas of the body. The pathogenesis of LELC remains unknown, but Epstein-Barr virus (EBV) has been shown to be strongly correlated with LELC at several anatomic sites, including the lungs and thymus. To the best of our knowledge, EBV-associated LELC has never been reported in both the posterior mediastinum and liver. Herein, we report the case of a 41-year-old female diagnosed with LELC in both the posterior mediastinum and liver and discuss whether it is beneficial to perform surgery on advanced LELC when resectable metastases are found.

**Patient concerns:**

The patient was a 41-year-old woman who had been suffering from intermittent pain in the upper right quadrant for 3 months without obvious cause and was admitted to our hospital with occasional nausea without vomiting.

**Diagnosis:**

Her cancer antigen 125 and cytokeratin 19 fragment levels were elevated, whereas alpha-fetoprotein and alanine aminotransferase were normal. Computed tomography (CT) and magnetic resonance imaging revealed a mass in the S6 segment of the liver. Whole-body positron emission tomography/computed tomography (PET/CT) revealed a 3.2-cm mass in the posterior mediastinum and a 6.7-cm mass on the right side of the liver. We made a diagnosis of LELC based on the histological and immunohistochemical findings of specimens obtained by operation. However, it was difficult to determine the primary origin of the tumor.

**Interventions:**

The patient underwent mediastinal tumor resection, hepatectomy, and diaphragmatic repair. Thereafter, she was administered paclitaxel and cisplatin as adjuvant chemotherapy.

**Outcomes:**

The postoperative course was uneventful, and the patient was discharged 10 days later. Although she was administered paclitaxel and cisplatin as adjuvant chemotherapy, we noted recurrence during the 4-month follow-up examination. Then, the patient passed away 5 months after surgery.

**Lessons:**

We present the first case of LELC found in both the posterior mediastinum and liver and describe the functionality of PET/CT for finding occult carcinomas and identifying their primary tumor origin. Additional studies are urgently needed to discover whether it is beneficial to perform surgery on advanced LELC when resectable metastases are revealed by PET/CT.

## 1 Introduction

Lymphoepithelioma-like carcinoma (LELC), which is a form of undifferentiated carcinoma characterized by abundant lymphoid components, was originally described in the nasopharynx.^[1]^ More recently, these tumors have been reported to occur in several organs, including the salivary glands,^[2]^ lungs,^[3]^ stomach,^[4]^ and liver.^[5]^ The thymus and lungs are the most common locations for primary mediastinal LELCs.^[6]^ However, an LELC originating from the posterior mediastinum has not been previously reported. Primary LELCs of the liver, which have only recently been recognized by the World Health Organization as a variant of hepatocellular carcinoma (HCC) (LEL-HCC), are also rare.^[7]^ The pathogenesis of LELC remains unknown, but Epstein-Barr virus (EBV) has been shown to be strongly correlated with LELC at several anatomic sites, including the lungs and thymus.^[8]^ Regardless of origin, the morphological features and prognosis of LELC appear to be the same based on the limited information that has been reported. To the best of our knowledge, LELC has never been reported in both the posterior mediastinum and liver. Herein, we report the case of a 41-year-old woman diagnosed with LELC in both the posterior mediastinum and liver, and we review the literature on treatments for LELC.

## 2 Case report

**Figure 1:**
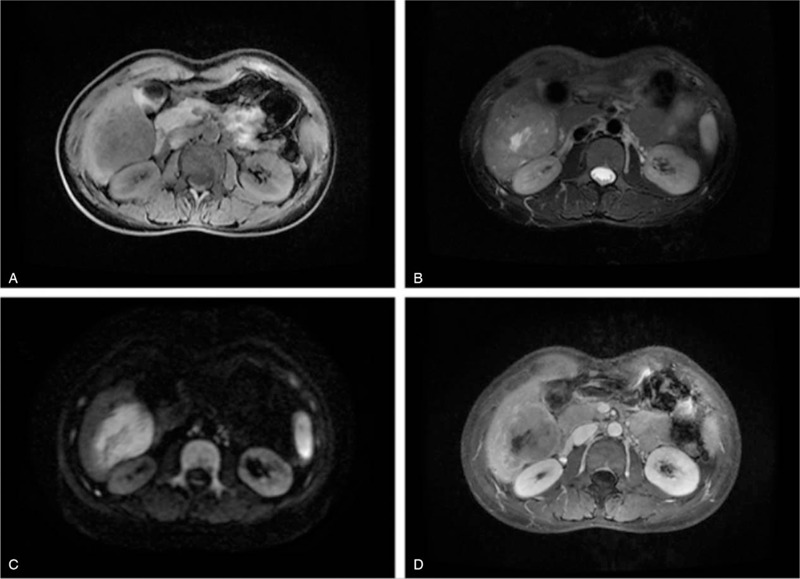
Magnetic resonance imaging (MRI) findings. A, On MRI, the mass in the right liver appeared hypointense on T1-weighted images. B, The mass appeared inhomogeneous hyperintense on T2-weighted images. C, The mass appeared inhomogeneous hyperintense on DWI-weighted images. D, On contrast-enhanced MRI, the mass was slightly enhanced.

**Figure 2:**
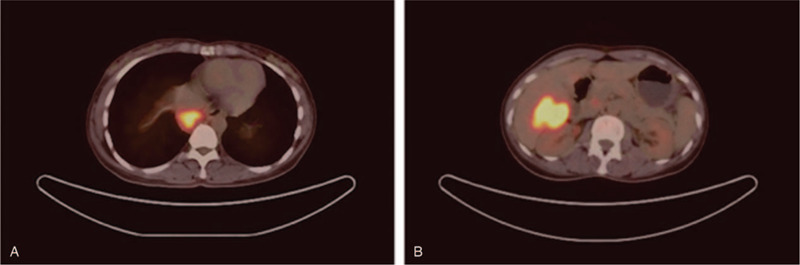
Positron emission tomography/computed tomography findings. A, The imaging revealed a 2.3-cm hypodense lesion in the posterior mediastinum, which shows significantly different levels of FDG uptake (SUV maxy ≈ 8.1). B, The imaging revealed a 6.7-cm hypodense lesion in the S6 lobe of the liver with remarkable FDG uptake (SUV max ≈ 8.1).

**Figure 3:**
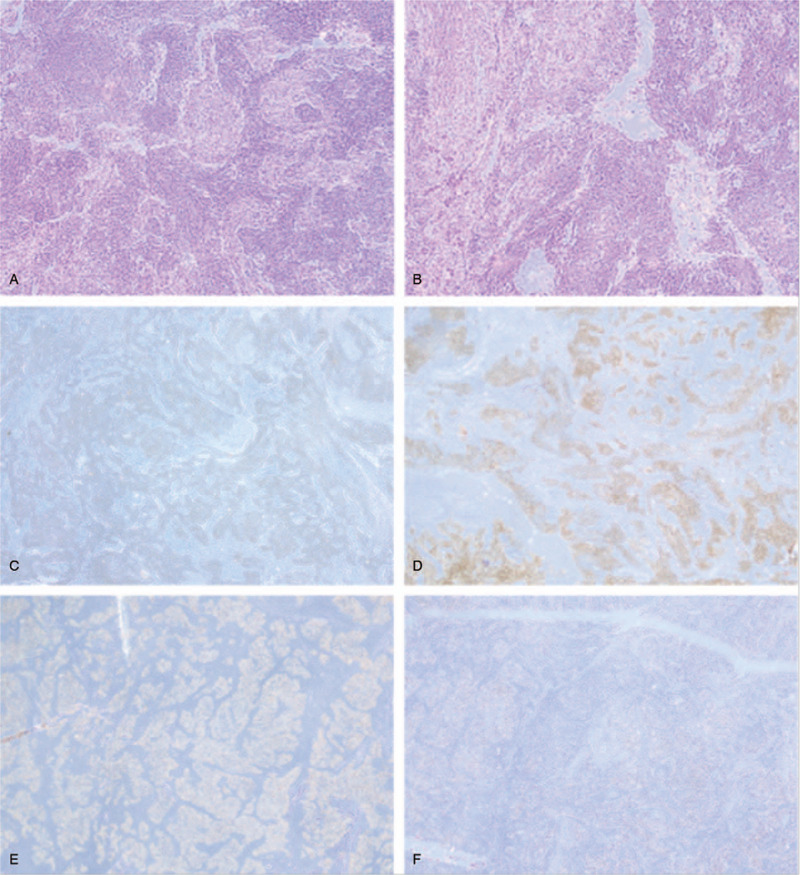
Microscopic examination (the posterior mediastinum specimen). Microscopically, in the solid tumor, abundant poorly differentiated, atypical, large epithelial cells with an eosinophilic cytoplasm were found (A, B, HE × 40). Immunohistochemically, CK (pan), CK (5/6), P63, and EBER (C, D, E, F × 100) were positive.

The patient was a 41-year-old woman who had been suffering from abdominal pain and nausea for 3 months before evaluation. She had experienced intermittent pain in the upper right quadrant for 3 months without obvious cause and occasional nausea with no vomiting but did not exhibit fever, diarrhea, or abdominal distension. She underwent ovarian cystectomy 10 years prior and had no history of infection with hepatitis B or C. Her other medical and family histories were unremarkable. She did not smoke and seldom drank. Abdominal palpation revealed mild pain lacking rebound tenderness in the upper right quadrant. The level of cancer antigen 125 (CA 125) was 65.2 U/mL (normal range 0.0–35.0 U/mL), and the level of cytokeratin 19 fragments (CYFRA 21-1) was 60.7 ng/mL (normal range 0.0–60.7 U/mL). Alpha-fetoprotein (AFP), total bilirubin, and alanine aminotransferase were within normal ranges. Enhanced abdominal computed tomography (CT) revealed a 6.7-cm hypodense lesion in the S6 segment of the liver. A 6.7 × 5.4-cm hepatic mass was found on magnetic resonance imaging (MRI). The T1 image showed a low signal, whereas T2 and diffusion-weighted imaging showed a high signal. The contrast-enhanced images showed a mixed signal (Fig. 1). A biopsy sample from the hepatic lesion indicated LELC with probable metastasis. She was referred for whole-body positron emission tomography/computed tomography (PET/CT), which revealed a 3.2-cm mass in the posterior mediastinum and a 6.7-cm mass on the right lobe of the liver, as well as lymphadenopathy in the hepatic portal region and posterior peritoneum (Fig. 2). The lungs and stomach, which are common sites for LELC, showed no signs of abnormality. After consulting with colleagues and taking all diagnostic findings into account, we decided that resection surgery of the LELC was the best course of action. We began resection surgery in cooperation with the doctors from the thoracic surgery department after obtaining informed consent from the patient and her relatives. We first found the hepatic tumor, which was dark red and measured approximately 8 × 6 cm, located in the S6 segment with clear boundaries. We separated the ligaments around the liver, dissected the first, second, and third hepatic hila, and began to completely remove the liver tumors along the 2 cm of the tumor boundary. After hemostasis was completed, the diaphragm was opened with an electric knife along the inferior vena cava, as planned. The mediastinal tumor (3 × 2 cm) lay behind the postcava with lymphadenopathy (1.5 × 1 cm). We found no other metastases, and there was no obvious adhesion between the tumor and the surrounding area. Thus, we finally performed mediastinal tumor resection, hepatectomy, and diaphragmatic repair. Microscopically, the neoplasms were full of poorly differentiated epithelial cells with eosinophilic nucleoli. The epithelial cells were surrounded by dense lymphoid stroma extending inside the tumor. The immunohistochemical analysis was CD3+, CD5+, CD20+, CD5/6+, P63+, CK-pan+, EBV-encoded RNA+ (EBER+), hepatocyte−, and AFP− (Fig. 3). Ki-67 was present in 40% of malignant cells. A diagnosis of LELC was made based on the histological and immunohistochemical findings, and the posterior mediastinum was determined to be the likely primary region of origin. The postoperative course was uneventful, and the patient was discharged 10 days later. Thereafter, she was administered paclitaxel and cisplatin as adjuvant chemotherapy, and we noted recurrence during the 4-month follow-up examination. The patient passed away 1 month later.

## 3 Discussion

LELC was initially discovered in the 1920s in the nasopharynx of patients infected with EBV.^[1]^ Since then, more cases originating from a variety of organs have been described, including the salivary glands and stomach. These tumors are characterized by undifferentiated carcinoma cells with abundant infiltrating lymphocytes.^[7]^ Although the pathogenesis of LELC remains unknown, it has been correlated with various viral infections, such as EBV in the lungs,^[9]^ HBV in the liver,^[10]^ and HPV in the genitals.^[11]^ An in situ hybridization (ISH) test for EBER is generally used to distinguish LELC from other lung cancers.^[9]^ A favorable prognosis has been proposed in EBV-associated LELC arising in the stomach, lungs, and uterine cervix.^[12–14]^ In our present patient, the EBER test was positive, indicating that LELC found in uncommon regions may also have close etiopathogenetic linkage with EBV infection.

The associations between serum tumor markers and LELC are still unclear. Elevated CA 125 can be found in adenocarcinoma and squamous cell carcinoma, whereas CYFRA 21-1 may be elevated in squamous cell carcinoma.^[15]^ We found elevated CA 125 and CYFRA 21-1 in our patient, which is consistent with a previous study that recommended the use of CA 125 and CYFRA 21-1 to monitor LELC activity and its response to treatment.^[16]^ On the contrary, the patient's serum levels of AFP and CA 19-9 were unremarkable, which may be helpful for distinguishing LELC from primary liver tumors, such as HCC and intrahepatic cholangiocarcinoma.^[17,18]^ LELC appeared similar to in situ malignant tumors on CT or MRI, which show fast inward and outward flow when contrasted. Park et al^[19]^ reported that F-18 FDG PET/CT shows high sensitivity for lymphoepithelioma-like gastric carcinoma as well as distant metastases, consistent with the findings in our patient whose 2 regions showed high sensitivity. PET/CT is thought to reliably detect occult carcinomas in different regions, and it allowed us to identify the primary tumor after the biopsy data raised our suspicions. Based on our case, we recommend the use of PET/CT when LELC is suspected in uncommon regions such as the liver.

The precise diagnosis was made by pathology, and the immunohistochemical findings showed abundant poorly differentiated atypical large epithelial cells with an eosinophilic cytoplasm and high expression of CK, CK5/6, and P63.^[20]^ We arrived at this patient's diagnosis by performing the liver biopsy first. Although it was difficult to determine the primary region of origin for this patient's tumor, we believe the diagnosis of posterior mediastinal LELC accompanied by liver metastasis is correct for the following reasons. First, LEL-HCC is quite rare, with only approximately 40 cases reported to date.^[21]^ In addition, HBV or HCV infection and liver cirrhosis are common in these tumors. The vast majority of hepatic cases are negative for EBV by ISH and sequencing,^[5]^ but LELC of the mediastinum is common. Moreover, the pathologist thought it was difficult to determine the region of LELC origin based on these uncommon regions. They found that there are no significant differences in cell morphology and immunohistochemistry between the 2 specimens and, combined with the similar imaging findings, believed that the mediastinum was more likely a primary site, and the locus in the liver was more likely to metastasize.

Standard treatments for LELC have not been established. Currently, first-line treatment of LEL-HCC includes liver resection and liver transplantation.^[21]^ Most patients with pulmonary lymphoepithelioma–like carcinoma (PLELC) undergo radical resection as the primary treatment when diagnosed at an early and resectable stage.^[22]^ A conservative management strategy for submucosal invasion treated by endoscopic submucosal dissection was considered for early stomach LELC.^[23]^ Although chemotherapy was thought to be a suitable treatment for patients with advanced stage PLELC, radiotherapy was more often used in breast LELC.^[24]^ Liang et al^[25]^ recommended platinum-based drugs combined with third-generation chemotherapy as first-line treatment for PLELC. In addition, some researchers have seen positive results using PD1/PD-L1-targeting immunotherapy for different types of LELC.^[26,27]^

Our patient was in an advanced stage, and the choice of treatment was challenging. The overall survival rate for patients with advanced LELC is low. Tay et al^[28]^ reported 5-year survival rates for advanced stage patients with PLELC in Singapore as less than 10%. Combination therapy is the best option to achieve the best possible outcome. Radical resection of hepatic metastases from colonic carcinoma has been shown to increase the 5-year survival rate from 0 to 5% to 25% to 40%.^[29]^ In a meta-analysis comparing the efficacy of surgical treatment with that of palliative care for 1147 patients with hepatic metastasis from pancreatic carcinoma, the 1-year survival rates were 52.8% and 27.1%, respectively, and the 3-year survival rates were 17.2% and 3.7%, respectively.^[30]^ For our patient, she was young, the focus was limited to 2 resectable sites, and the efficiency of nonsurgical treatment, such as chemotherapy or radiotherapy, was not satisfactory. To improve her treatment outcome, we opted to perform the surgery in coordination with doctors from the thoracic surgery department, as the tumor was well encapsulated and did not have microvascular invasion. Whether postoperative chemotherapy is effective in cases of LEL-HCC remains unknown due to a lack of data. Given the immunohistochemical finding that Ki-67 staining was present in more than 40% of cells, we chose paclitaxel and cisplatin as adjuvant chemotherapy for our patient to reduce the risk of recurrence. Unfortunately, we noted recurrence after 4 courses of adjuvant chemotherapy over a period of 4 months. More studies on advanced LELC with resectable metastasis are necessary to determine whether postoperative chemotherapy is beneficial.

## Author contributions

**Conceptualization:** Xiao-Hui Qian, Dong-Kai Zhou.

**Supervision:** Wei-Lin Wang and Dong-Kai Zhou.

**Writing – original draft:** Xiao-Hui Qian.

**Writing – review & editing:** Wei-Lin Wang.
